# Pathogen identification and fungicide screening of Sugarcane White Rash Disease in China

**DOI:** 10.3389/fmicb.2026.1860711

**Published:** 2026-06-22

**Authors:** Qian Shi, Zhenxin Huang, Haohua Huang, Rui Yang, Xiaoyi Mo, Jiaorong Meng

**Affiliations:** 1 Guangxi Key Laboratory of Sugarcane Biology, College of Agriculture, Guangxi University, Nanning, Guangxi, China; 2 State Key Laboratory for Conservation and Utilization of Subtropical Agro-Bioresources, Nanning, Guangxi, China; 3 Ministry and Province Co-sponsored Collaborative Innovation Center for Sugarcane and Sugar Industry, Guangxi University, Nanning, China

**Keywords:** chemical control, *Elsinoë sacchari*, fungicide, phylogeny, sugarcane white rash disease

## Abstract

Sugarcane is the most important sugar crop in China. In recent years, a disease characterized by white rash symptoms has become endemic in sugarcane fields in the coastal area of Guangxi Province, China. A fungal species was consistently isolated from diseased leaves of sugarcane plants, and the pathogenicity assays were consistent with its etiology, reproducing symptoms identical to those observed in the field. Based on host range, symptomatology, and morphological characteristics, the causative agent was identified as *Elsinoë sacchari*. The fungus grew fastest on potato dextrose agar (PDA) at pH 6.0 and 25 °C, and grew well in a medium with oat as the carbon source or ammonium sulfate as the nitrogen source. *In vitro* fungicide sensitivity assays identified Difenoconazole as the potent compound for inhibiting mycelial growth and Pyraclostrobin·boscalid as the potent compound for inhibiting spore germination. Field trials showed that application of 75% Trifloxystrobin·tebuconazole at 0.250 g/L resulted in the lowest disease severity, while application of 38% Pyraclostrobin·boscalid at 0.190 g/L or 75% Trifloxystrobin·tebuconazole at 0.250 g/L yielded the highest cane production. This study clarified the key biological characteristics of *E. sacchari* and successfully screened several fungicides suitable for field control of Sugarcane White Rash Disease.

## 1 Introduction

Sugarcane is a perennial monocotyledonous C4 plant of the gramineous family, known for its high photosynthetic efficiency (Ali et al., 2019). As the most important sugar crop in China, sugarcane accounts for about 90% of the total sugar production. Guangxi Province is the largest sugarcane production base in China (Yang et al., 2021). Fungal disease has been an important limiting factor that negatively impacts cane yield and sugar content in sugarcane (Amaresh et al., 2025). Furthermore, pathogenic microorganisms can accumulate within sugarcane, leading to the degradation of sugarcane varieties (Bagyalakshmi et al., 2019). The incidence of sugarcane diseases is increasing at an alarming rate, and a considerable share of sugar production is lost every year due to these diseases (Viswanathan and Rao, 2011). Except for the smut fungus, the majority of fungal pathogens cause foliar disease in sugarcane (Selvakumar et al., 2023). Among these, *Elsinoë sacchari* (asexual stage: *Sphaceloma sacchari*) causes white rash lesions on sugarcane leaves, termed Sugarcane White Rash Disease (SWRD). This disease was first reported in Taiwan in 1957 (Lo, 1957; Pelegrín et al., 2012). Based primarily on symptom similarity, it has also been reported to occur in sugarcane plantations in the provinces of China, Guangdong and Guangxi (Wei et al., 2012). Despite a long history of this disease, the biology of the causal agent remains poorly understood.

*Elsinoë* species infect the leaves and fruits of various important crops and trees, causing scab-like lesions or corky tumor-like outgrowths. These infections often lead to premature defoliation and yield reduction, as seen in citrus scab, grape black pox, peanut scab, and willow leaf scab disease (Hyun et al., 2001; Jayawardena et al., 2015; Poolsawat et al., 2008; Su et al., 2022; Zhao et al., 2018). *Elsinoë* species grow slowly on artificial media, forming fleshy, wrinkled, and variably colored colonies, which makes their isolation and study challenging (Chung, 2011; Masheti Y. O. et al., 2024).

In July 2022, a SWRD like disease occurred in a village of Hepu County, Guangxi Province, China, in the coastal area of the Beibu Gulf region. A comprehensive study was subsequently conducted to identify the causal agent and characterize its cultural characteristics. The study also aimed to evaluate the efficacy of various fungicides for potential field control of this disease.

## 2 Materials and methods

### 2.1 Field survey and isolation of the pathogen

A field survey was conducted between May and July 2022 in Xichang Town, Beihai City, Guangxi Province, using a five-plot sampling method (Zou et al., 2011). Leaf samples were collected from sugarcane plants showing SWRD-like symptoms and kept in sealed plastic bags. Samples were subjected to fungal isolation within 48 h of collection.

For isolation, leaf tissues at the junction of diseased and healthy regions were cut into approximately 0.3 cm × 0.3 cm squares and soaked in 75% alcohol for 30 s, followed by surface sterilization in 5% NaClO for 4 min, rinsed 3 times with sterile water, then placed on a sterilized filter paper to remove residual solution (Huang et al., 2023). The sterilized tissues were transferred onto a potato dextrose agar (PDA, Beijing Coolaber Technology, Beijing, China) plate supplemented with 100 μg/mL streptomycin and incubated at 25 °C. Mycelium that emerged from the tissue edges was then sub-cultured onto a fresh PDA plate with streptomycin. After purification by single-spore isolation, pure cultures were preserved in 20% glycerol and stored at 4 °C for further studies.

### 2.2 Cultural conditions

To determine the optimal conditions for fungal growth, the effects of various media, carbon and nitrogen sources, temperature, pH, and light were evaluated. Nine different media were tested: PDA, corn meal agar (CMA: corn flour 30.0 g, agar 18.0 g, distilled water to 1.0 L), sugarcane-leaf-decoction saccharose agar (SSA: the fresh sugarcane leaves were 200.0 g, boiled with water for 30 min, filtered, and the filtrate was added with 20.0 g sucrose, 20.0 g agar, and distilled water to 1 L), Czapek-Dox agar (NaNO_3_ 2.0 g, KH_2_PO_4_ 1.0 g, KCl 0.5 g, MgSO_4_·7H_2_O 0.5 g, FeSO_4_ 0.01 g, sucrose 30.0 g, agar 18.0 g, distilled water to 1.0 L), potato sucrose agar (PSA: potato flour 6.0 g, sucrose 15.0 g, agar 18.0 g, distilled water to 1 L), minimal medium (MM: Sucrose 30.0 g, KH_2_PO_4_ 1.0 g, MgSO_4_·7H_2_O 0.5 g, KCl 0.5 g, FeSO_4_ 0.01 g, NaNO_3_ 2.0 g, trace element 1 mL, agar 16.0 g, distilled water to 1.0 L), oat agar (OA: oatmeal 30.0 g, distilled water to 1.0 L, heated in boiling water bath for 1 h, filtered with four layers of gauze, filtered to a constant volume of 1.0 L, agar 18.0 g), and V8 and malt extract agar (MEA: malt extract 25.0 g, agar 18.0 g, distilled water to 1.0 L) (Huang et al., 2023; Li and Nan, 2009; Luiz and Keith, 2021; Li et al., 2022). For the carbon and nitrogen source assay, Czapek-Dox agar was used as the basal medium, in which the original carbon or nitrogen source was replaced with the carbon or nitrogen source under test.

The effects of temperature, pH, and illumination on the mycelial growth were also analyzed (Shao et al., 2024; Wang et al., 2023). The mycelial plugs were inoculated at the center of PDA medium and incubated at 5, 10, 15, 20, 25, 28, 30, 35, and 40 °C. To evaluate pH effects, the pH of the PDA medium was adjusted with HCl and NaOH to values of 2, 4, 5, 6, 7, 8, 10, and 12 prior to inoculation. Various light/dark periods were used to test the optimal conditions for the fungus’s growth and development. All treatments were performed with four replicates.

### 2.3 Pathogenicity assay

Seedlings derived from the cane cuttings of the sugarcane variety Zhongzhe 9 were grown in greenhouse pots at a temperature ranging 25–28 °C. Five-week-old plants at the 5-leaf stage were used for the pathogenicity assay. A spore suspension containing 1 × 10^6^/mL and 0.1% Tween-80 was sprayed onto the leaves of the sugarcane seedlings (5 mL per plant, 5 leaves; 5 plants per treatment). An equal amount of distilled water containing 0.1% Tween-80 was used as the control (Huang et al., 2023). Ten sugarcane plants were inoculated with each treatment. After inoculation, the plants were covered with black plastic bags to maintain high humidity for 3 days, then kept in a greenhouse. The plastic bags were removed after 3 days. Disease development was observed and photographed at 5, 10, 20, 30, 40, and 60 days post-inoculation (dpi). Diseased leaves were collected, and the fungal pathogen was re-isolated for characterization. Additionally, pathogenicity of the isolates against peanut, rice, wheat, sorghum, and foxtail millet was assessed using the same method described above.

### 2.4 Morphological characterization of the fungus

The single spore-purified fungal strains were cultured on PDA plates in the dark at 25 °C for morphological observation, including colony morphology and sporulation. OA medium was used to induce sporulation. The morphology was observed under a light microscope (Olympus BX 53, Tokyo, Japan). The morphology, color, and conidia size (100 replicates) were observed, measured, and photographed (Santos et al., 2018). Colony images were captured using a digital camera (Canon EOS 80D 18–200 mm, Tokyo, Japan).

### 2.5 Molecular identification of the fungus

Total genomic DNA was extracted from mycelium cultured for 15 days using a previously described method (Raeder and Broda, 1985) and stored at −20 °C. The ribosomal internal transcribed spacer (ITS), mitochondrial large subunit ribosomal RNA (rRNA) (LSU), RNA polymerase II (RPB2), and the translation elongation factor 1-*α* (TEF1-α) genes were used for molecular identification of the pathogen (Jayawardena et al., 2015). A polymerase chain reaction (PCR) was performed using the primers for ITS (ITS1 and ITS4) (Hyun et al., 2009), LSU (LR0R and LR5) (Sung et al., 2007); RPB2 (RPB2-5F2 and fRPB2-7cR) (Liu et al., 1999; Sung et al., 2007) and TEF1-α (elongation-1-F and elongation-1-R) (Hyun et al., 2009) to amplify ITS, LSU, RPB2, and TEF1-α, respectively. Each 25 mL amplification reaction system included 12.5 μL of 2 × Rapid Taq MasterMix (Vazyme Biotech, Nanjing, China), 1 μL fungal DNA (50 ng/μL), 1 μL of each primer (10 pmol), and 9.5 μL of ddH_2_O. The PCR was performed in a ProFlex 3 × 32-Well PCR System (Life Technologies, Singapore) under the following thermal profile: pre-denaturation at 94 °C for 3 min; denaturation at 94 °C for 30 s, annealing at 54 °C for 30 s (ITS 54 °C, LSU, RPB2 58 °C and TEF1-α 60 °C), extension at 72 °C for 45 s, 35 cycles, with a final extension at 72 °C for 10 min.

The PCR products were separated by electrophoresis on a 1.5% agarose gel in TBE buffer for 20 min, and the agarose gel DNA fragments were recovered and purified using the HiPure gel Pure DNA Mini Kit (Magen Biotech, Guangzhou, China) according to the manufacturer’s protocol. The purified PCR product was cloned into the pEASY-T1 vector (TransGen, China). For each gene and isolate, three positive transformants containing PCR products were selected and submitted to a professional sequencing service provider (Augct Biotech, Wuhan, China) for Sanger sequencing. The resulting sequences were compared with the reference sequences of ITS, LSU, RPB2, and TEF1-α of *Elsinoë* species retrieved from the GenBank database using the basic local alignment search tool for nucleotide (BLASTn) algorithm and refined with MEGA 11.0 software (Tamura et al., 2021). A multi-gene phylogenetic tree was constructed using the Neighbor-joining method with 1,000 bootstrap replicates (Rusinko and Mcpartlon, 2017).

### 2.6 Screening of potent fungicides *in vitro*

Sensitivity to commonly used fungicides (Supplementary Table S1) was evaluated by measuring inhibition of mycelial growth and conidial germination (Shin et al., 2017). For the mycelial growth assay, fungicides at varying concentrations were added to the PDA medium at approximately 50 °C and fully mixed, then poured into 9-cm-diameter Petri dishes, with an equal volume of sterile water added as a control. A fresh 6 mm diameter mycelial plug was inoculated onto the center of a fungicide-amended plate. The inoculated plates were incubated in the dark at 25 °C for 21 days. Each treatment was repeated 4 times. For the conidial germination assay, 100 μL of conidial suspension containing 1,000 spores was smeared onto a fungicide-containing PDA plate. The inoculated plates were incubated in the dark at 25 °C. When the germination rate in the control treatment reached approximately 90%, the number of spore germinations for each treatment was counted. The experiments were repeated 3 times.

Four randomly selected isolates were tested for sensitivity to the following fungicides: Trifloxystrobin·tebuconazole, Myclobutanil, Pyraclostrobin·boscalid, and Thiophanate-methyl, using both the mycelial growth and spore germination methods described above.

### 2.7 Field trial of Sugarcane White Rash Disease control by fungicides

Fungicides that showed strong inhibition of both mycelium growth and spore germination *in vitro* were selected for field trial of SWRD control, including 25% Myclobutanil at 4,000-fold dilution (0.0625 g/L) and 3,000-fold dilution (0.0833 g/L), 75% Trifloxystrobin·tebuconazole at 4,000-fold dilution (0.188 g/L) and 3,000-fold dilution (0.250 g/L), 38% Pyraclostrobin·boscalid at 2,000-fold dilution (0.190 g/L) and 1,500-fold dilution (0.253 g/L), and 70% Thiophanate-methyl at 1,000-fold dilution (0.700 g/L) and 750-fold dilution (0.933 g/L). These were used for the field control trial, with an equal volume of water applied as a control. The trials were conducted in 2023 in Xipo Village, Hepu County, Guangxi Province (108.57° E, 21.39° N) using the first ratoon of the sugarcane variety Guiliu 05136. Field plots measuring 6 m × 3 m were completely randomized with three replicates for each fungicide treatment. In each plot, 20 sugarcane plants were continuously monitored, and signs were affixed to record their serial numbers for future field investigations.

**Figure 1:**
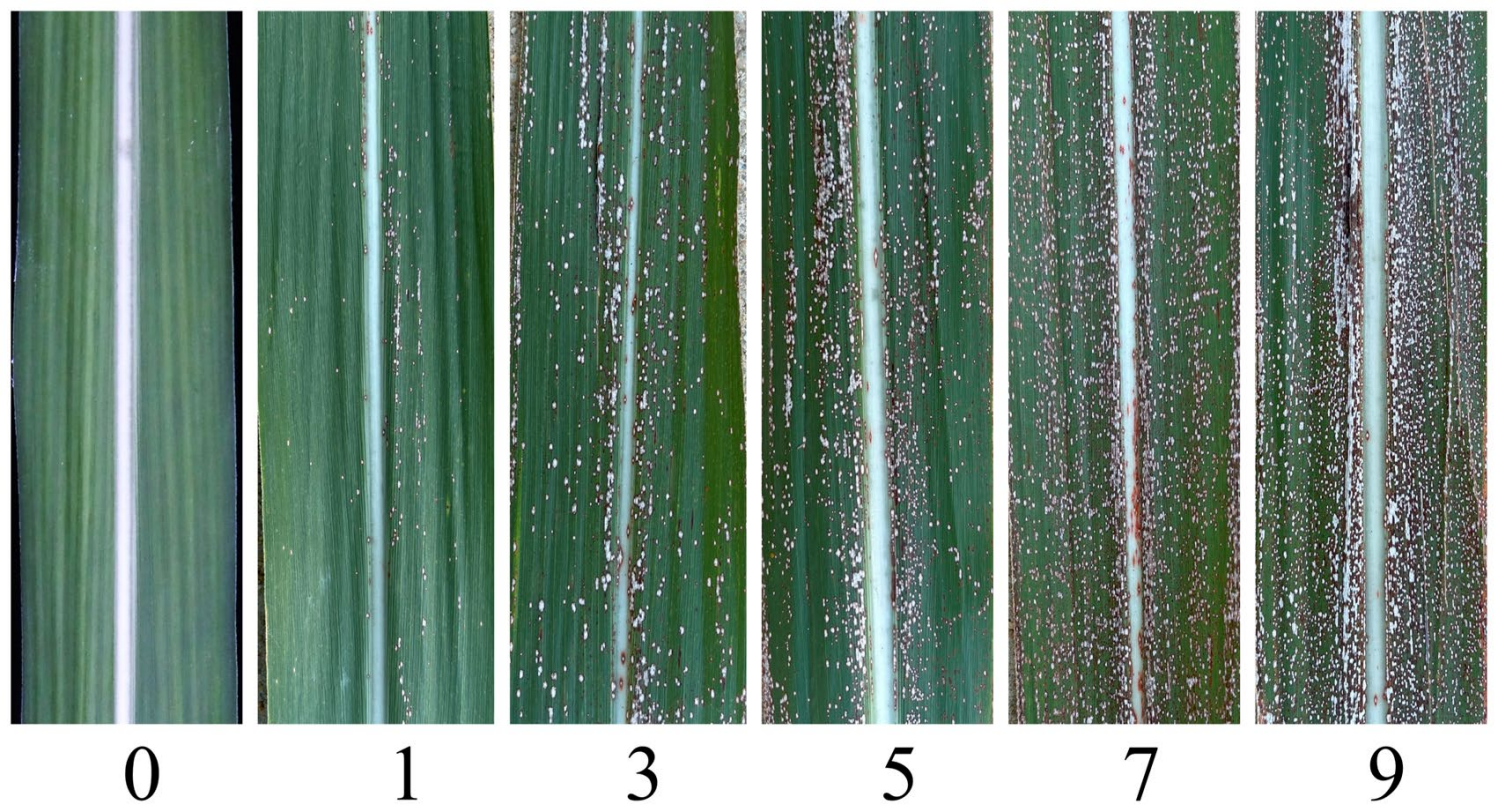
Schematic diagram of sugarcane white rash disease grading standard. Grade 0, no lesion on a leaf; Grade 1, lesion area accounting for less than 5% of the leaf area; Grade 3, lesion area accounting for 5-25% of the leaf area; Grade 5, lesion area accounting for 25-50% of the leaf area; Grade 7, lesion area accounting for 50-75% of the leaf; Grade 9, lesion area accounting for more than 75% of the leaf area.

The first spray was applied when the disease index (DI) reached approximately 15. A total of three sprays were applied with an interval of seven days (Dang et al., 2023) (Table 1). Disease progression was assessed on the same 20 tagged stalks in each plot. The disease severity of SWRD was classified using an arbitrary symptom scale (Figure 1). Accordingly, disease index (DI), disease index growth rate (DIGR), and disease relative control efficacy (DRCE) were calculated using the following formulas:


$$\mathrm{DI}=\frac{\sum (\text{Number of disease}\;\mathrm{at}\;\text{each level}\times \text{relative disease grade})}{\text{Total number of leaves investigated}\times 9}\times 100$$

$$\text{DIGR}\;(\%)=\frac{{\mathrm{DI}}_{\text{after}}-{\mathrm{DI}}_{\text{before}}}{{\mathrm{DI}}_{\text{before}}}\times 100$$

$$\text{DRCE}\;(\%)=\frac{{\mathrm{DI}}_{\text{before}}\times (1+{\text{DIGR}}_{\text{control}})-{\mathrm{DI}}_{\text{after}}}{{\mathrm{DI}}_{\text{before}}\times (1+{\text{DIGR}}_{\text{control}})}\times 100$$

where DI_before_ = DI before application of the fungicide, DI_after_ = DI after application of the fungicide, and DIGR_control_ = DIGR in control plots.

**Table 1:** Details of the application and investigation.

Treatment	Application time			Investigation time	
Water (CK)	June 7	June 14	June 21	June 5	July 1
Fungicide	June 7	June 14	June 21	June 5	July 1

### 2.8 Data analysis

All data were statistically analyzed using the Statistical Package for the Social Sciences (SPSS) version 26.0 software (SPSS, Inc., Chicago, IL, United States). The independent variable x was represented by the natural logarithms of the concentrations of each agent. In contrast, the dependent variable y was represented by the Probit values of the percentage of mycelial inhibition. The efficacy regression equation and the correlation coefficient (R^2^) were computed, along with the effective concentration at which 50% inhibition occurs (half maximal effective concentration [EC_50_]). One-way analysis of variance (ANOVA) and Duncan’s test (*p* = 0.05) were employed to determine significant differences among the treatments. The data were represented as mean ± standard deviation.

## 3 Results

### 3.1 Symptoms and incidence of the disease

**Figure 2:**
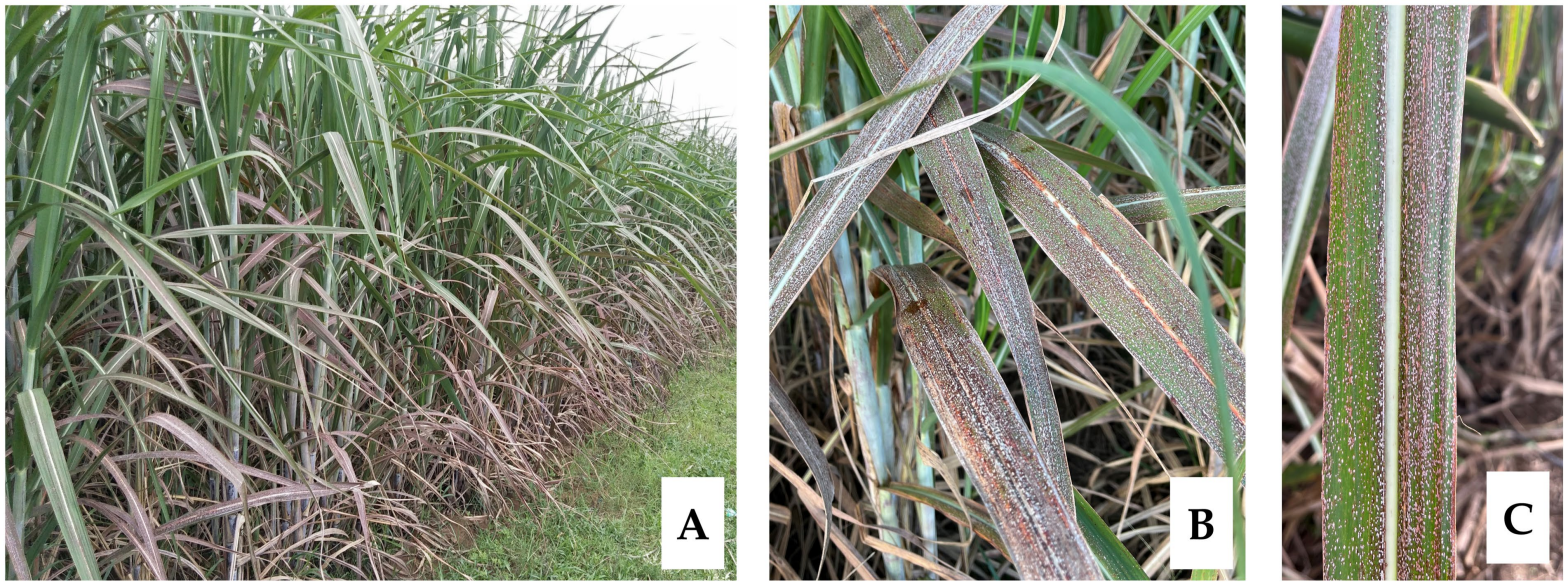
Symptoms of sugarcane white rash disease in the field. **(A)** Overview of the diseased sugarcane field, where a large area of plants shows symptoms, with yellowing and wilting commonly observed on the middle and lower leaves; **(B,C)** Typical symptoms of leaves, with numerous grayish-white lesions and narrow pinkish-white stripes on the surface, leading to leaf death in severe cases.

A field survey was conducted in the sugarcane planting area of Xipo Village, Hepu County, Guangxi Province, from May 2022 to July 2022, to investigate a disease with symptoms resembling previously described SWRD (Chen, 1982). The first symptom was tiny yellow, oval spots on the upper surface or midrib of the leaves, which then turned grayish-white, within approximately 1 week to 10 days. As the disease progressed, spots might merge to form narrow, pinkish-white stripes, ultimately leading to leaf death in severe cases (Figure 2). The outbreak of the disease was observed in early to mid-July. The disease incidence reached about 95% in the fields surveyed in early July.

### 3.2 Pathogen isolation and identification

**Figure 3:**
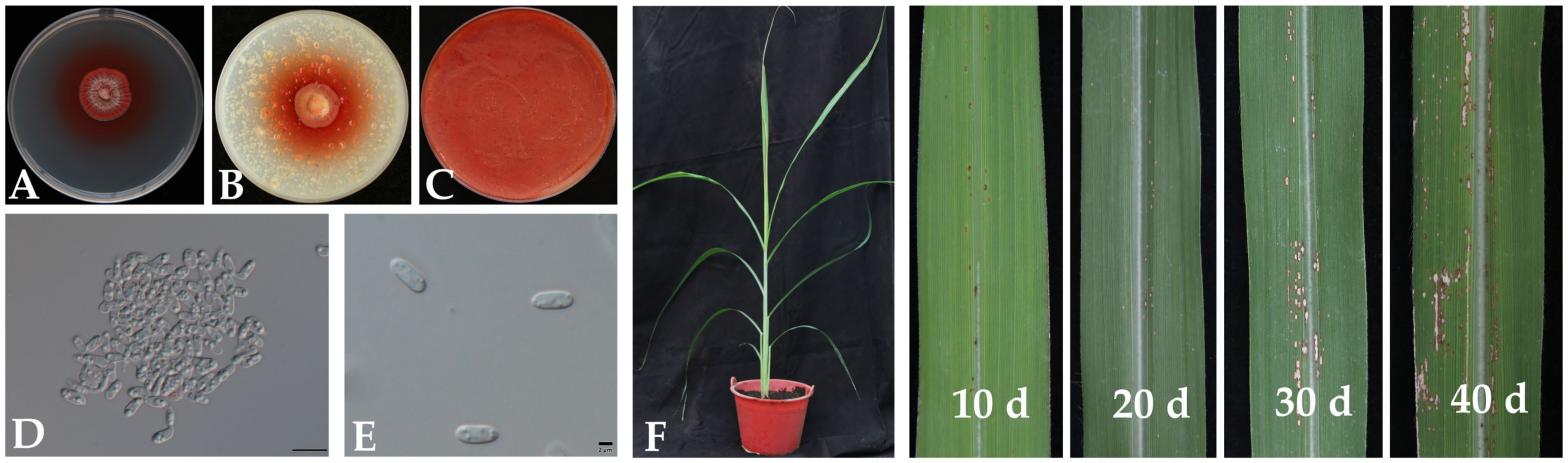
Morphological characteristics and pathogenicity assay of isolate EH036. **(A)** Colony morphology on PDA, 21 days post inoculation; **(B)** colony morphology on OA, 21 days post inoculation; **(C)** front views of the OA plate smeared with conidia after incubation for 10 days; **(D)** hyaline conidia; **(E)** hyaline conidia in higher magnification; **(F)** symptom development on sugarcane leaves inoculated with conidia from OA plates. Bars represent 10 μm in **(D)** and 2 μm in **(E)**.

Forty fungal isolates (EH001 to EH040) exhibiting typical *Elsinoë* characteristics, i.e., wrinkle colony, slow growth on PDA, and secretion of abundant red pigment into the medium (Figure 3A), were recovered from 100 SWRD-like leaf samples. The representative isolate EH036 showed poor sporulation on artificial culture media, and no spores were observed after 21 days of cultivation on PDA, MEA, and V8 media. We initially employed methods used for the induction of conidia for citrus scab pathogens *E. fawcettii* and *E. australis* (Hyun et al., 2015; Whiteside, 1975), but failed to induce conidia in *E. sacchari*. These isolates were able to produce conidia on OA medium (Figure 3B), though the conidia yields remained low (approximately 4.3 × 10^3^ conidia/plate). We compared inoculation of OA plates with fragmented mycelium from PDA colonies and with spores from OA colonies and found that spore inoculation yielded much more progeny conidia (about 203-fold). Subsequently, conidia were harvested by washing with sterile water to create a spore suspension, adjusted to a concentration of 10^6^ spores/mL, and then a 100 μL aliquot was smeared onto an OA plate and incubated at 25 °C in the dark. After 10 days, the plates were fully covered with merged colonies (Figure 3C), from which abundant conidia, about 8.09 × 10^7^/plate, were recovered. These conidia were all hyaline conidia, measuring 6.74 ± 0.37 μm in length and 2.89 ± 0.23 μm in width (Figures 3D,E).

Inoculation of the representative isolate EH036 onto leaves of the sugarcane variety Zhongzhe 9 reproduced symptoms that resembled those observed in the field (Figure 3F). Red spots appeared on the leaves 10 days after inoculation. On the 20th day, the lesions gradually turned from red to grayish-white. On the 30th day, the white spots continued to expand, and some lesions merged, forming narrow white stripes. On the 40th day, numerous lesions appeared, spreading throughout the infected area. The same fungus was re-isolated from the symptomatic leaves. The conidial morphology observed on naturally infected leaves was identical to that from experimentally inoculated leaves. After re-isolation from inoculated diseased leaves, the resulting isolate was cultured and shown to have hyphal and conidial morphological characteristics identical to those of the original inoculated strain EH036, thereby fulfilling Koch’s postulates. The isolate EH036 exhibits non-pathogenicity toward peanuts, rice, wheat, sorghum, and millet. Based on the host range, symptom expression, and morphological characteristics, EH036 was identified as *E. sacchari*.

**Figure 4:**
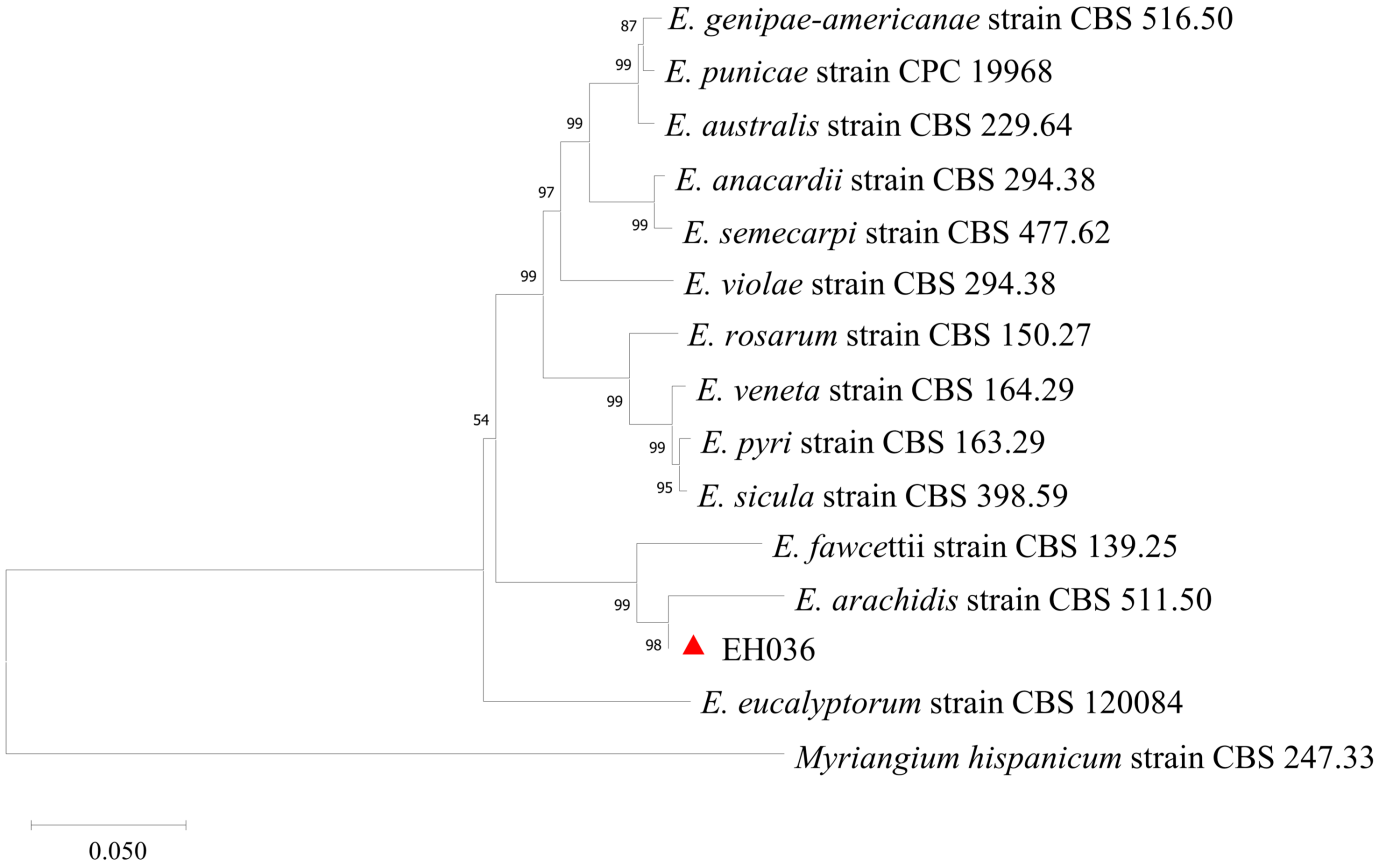
*Elsinoë* phylogenetic tree constructed with concatenated ITS, LSU, RPB2, and TEF1-α sequences. Other corresponding sequences were downloaded from the GenBank database.

To identify the causative agent, genomic DNA was extracted from the original isolate EH036 for molecular identification. The ITS, LSU, RPB2, and TEF1-α genes were amplified and sequenced. The resultant sequences ITS (PQ329350.1), LSU (PQ329351.1), RPB2 (PQ327936.1), and TEF1-α (PQ327935.1) were used to blast against the GenBank database. The highest scoring match sequences were from *E. australis* (ITS, 89.62% identity), *E. arachidis* (LSU, 98.60% identity), *E. fawcettii* (TEF1-α, 93.73% identity), and *E. phaseoli* (RPB2, 82.65% identity). Because no molecular sequences of *E. sacchari* were available in the database, sequences from other *Elsinoë* species were used to construct a phylogenetic tree (Fan et al., 2017; Jayawardena et al., 2015). Phylogenetic tree constructed with concatenated ITS-LSU-RPB2-TEF1-α showed that EH036 clusters closely with *E. arachidis*, the pathogen responsible for peanut scab, among many other species of *Elsinoë* (Figure 4). Thus, EH036 was identified as a species of the *Elsinoë* genus. The sequences of the ITS, LSU, RPB2, and TEF1-α gene regions were further compared between EH036 and *E. arachidis*. As shown in Supplementary Figure S1, sequence divergences of the ITS and LSU between EH036 and *E. arachidis* were 20% and 1.8%, respectively, greater than the thresholds suggested for discrimination of filamentous fungal species (Vu et al., 2019). Additionally, divergences of 23.1% in RPB2 and 26.6% in RPB2 were observed, further supporting the distinction between the two isolates.

### 3.3 Growth characteristics of *Elsinoë sacchari* under various cultural conditions

**Figure 5:**
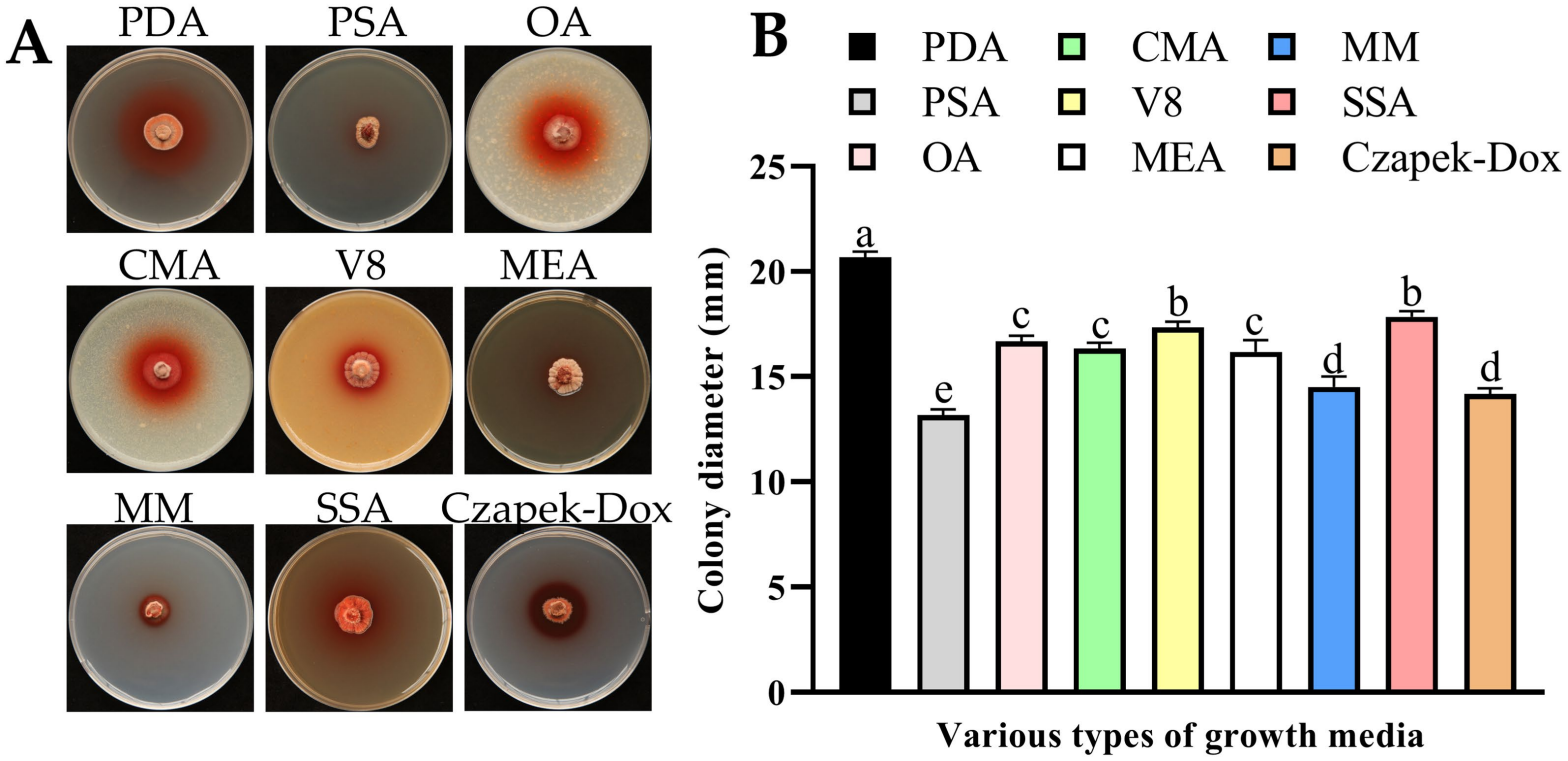
Growth of EH036 on different media. **(A)** Photographs of colonies on various media; **(B)** Colony diameters on different media. Cultures were kept at 25°C in dark. Photos were taken 21 days post inoculation. Columns with the same letters mean no significant difference according to Duncan’s test (*p* < 0.05).

A total of nine media, including PDA, PSA, OA, CMA, V8, MEA, MM, SSA, and Czapek-Dox, were evaluated for their suitability for culturing *E. sacchari* EH036. The fungus grew fastest on PDA, at 0.6 cm in diameter per week, reaching a colony diameter of 20.70 mm after 21 days. The colonies were petal-shaped with irregular edges, often displaying surface wrinkles radiating from the center, covered with either sparse or dense white, fluffy aerial hyphae, and with reddish to brown pigment diffused into the medium. However, no spores were produced. Similarly, wrinkled colonies with white or red aerial mycelium and red pigmentation were seen on PDA, V8, and SSA plates. Colonies on PSA and MEA were wrinkled with notably decreased pigmentation. In contrast, colonies grown on MM and Czapek-Dox agar were flat with much less aerial mycelium (Figure 5). Among these media, conidial production was observed only on OA plates. These media exhibited significant differences in nutritional composition. Specifically, PDA, V8, and OA are nutrient-rich complex media formulated with plant-derived extracts, whereas MM and Czapek-Dox are chemically defined synthetic media containing only single pure carbon and nitrogen sources without any organic complex additives. *E. sacchari* EH036 exhibited a clear vegetative growth preference for nutrient-rich complex media.

**Figure 6:**
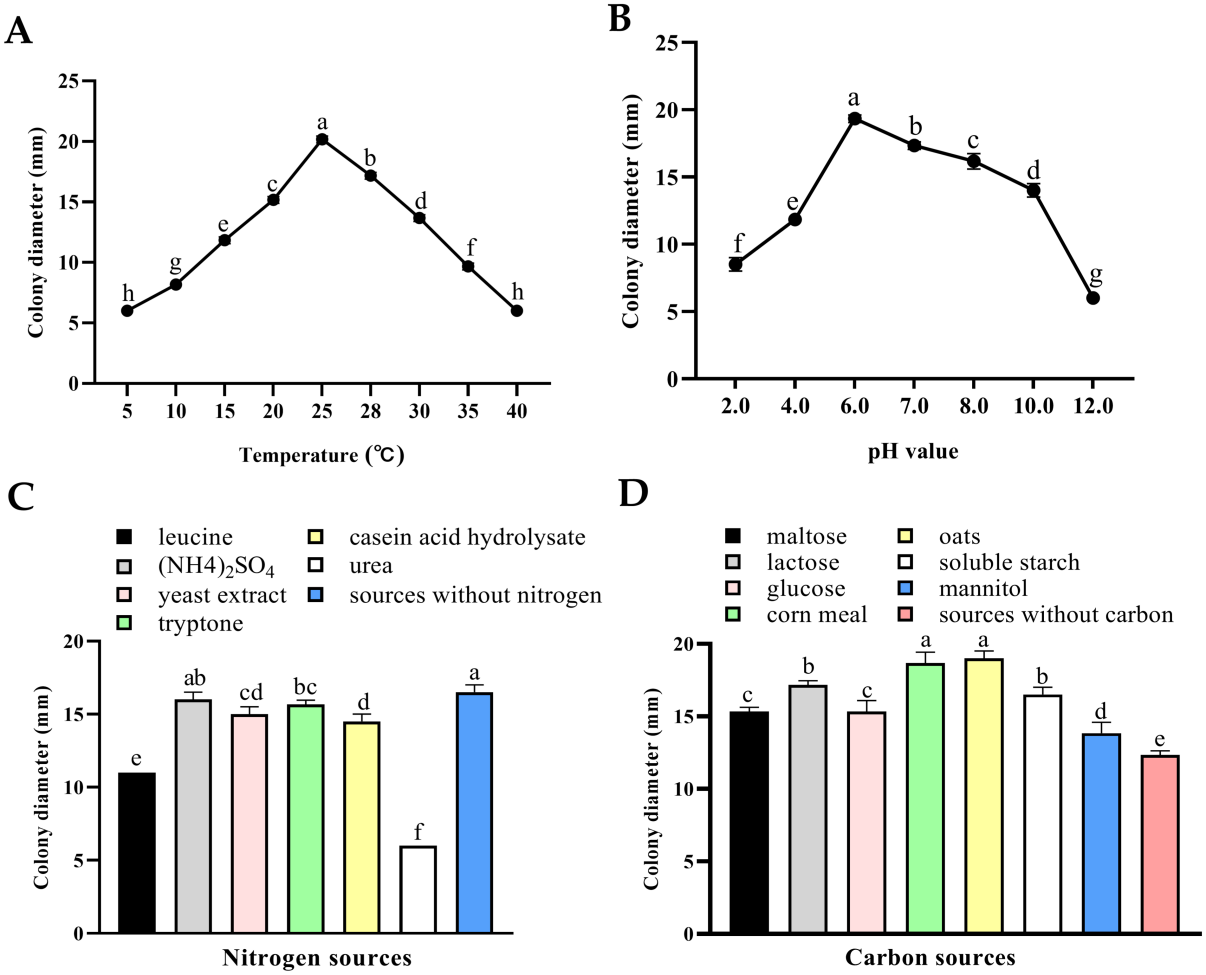
Effect of temperature, pH, nitrogen, and carbon on the growth of the EH036. **(A,B)** Used PDA medium as the test medium; **(C,D)** used Czapek-Dox agar medium as the replacement medium. **(A)** Effect of temperature on mycelial growth; **(B)** effect of pH on mycelial growth; **(C)** effect of different nitrogen sources on mycelial growth; **(D)** effect of different carbon sources on mycelial growth. The error bar is shorter than the symbol size and will not be displayed. Columns with the same letters mean no significant difference according to Duncan’s test (*p* < 0.05).

The EH036 grew on PDA of temperatures ranging from 5 °C to 40 °C, with the optimal temperature at 25 °C, and the colony diameter after 21 days was 20.17 mm (Figure 6A). Extreme temperatures have a significant impact on hyphal growth. When the temperature was lower than 15 °C or higher than 30 °C, mycelial growth was inhibited. Mycelium growth stops at 5 °C and 40 °C. The isolate EH036 showed a strong ability to adapt to pH and grew under conditions ranging from pH 2.0 to pH 12.0, but grew slowly under any extreme conditions. The optimal pH was 6.0, and the colony diameter after 21 days was 21.33 mm. This fungus prefers a slightly acidic to neutral environment (Figure 6B).

The EH036 could utilize a variety of nitrogen sources. The fastest colony growth was observed on the nitrogen-free medium, where the diameter reached 15.67 mm after 21 days, although the mycelium was sparse. The second fastest growth was on plates with ammonium sulfate as the sole nitrogen source, reaching a diameter of 15.00 mm after 21 days. On media with urea as the nitrogen source, the colonies ceased growing. The slowest growth was on medium with urea as the sole nitrogen source, followed by on medium with leucine as the sole nitrogen source (Figure 6C). It was noted that colonies exhibited dense aerial hyphae on media containing ammonium sulfate or yeast extract as the sole nitrogen source, suggesting healthy conditions. Moreover, a viscous substance on the colony surface was observed on medium containing tryptone or acid-hydrolyzed casein as the sole nitrogen source.

The EH036 could grow with all tested carbon sources and grew relatively well on media with oat or corn meal as the sole carbon source, with colonies reaching 18–19.00 mm in diameter after 21 days (Li et al., 2018). In contrast, the smallest colony was recorded on the carbon-free medium, on which the mycelium was sparse. Colony diameters of 13.5–17 mm were seen when the fungus was grown on media with mannitol, maltose, glucose, soluble starch, and lactose as the sole carbon source (Figure 6D). Compared with the carbon-free medium, all carbon-source-substituted media displayed higher colony growth rates. Of note, sparse mycelia were formed on media with lactose and mannitol as carbon sources (Huang et al., 2023).

### 3.4 Screening of potent fungicides for inhibition of *Elsinoë sacchari*

A total of 15 fungicides were evaluated for their inhibitory effects on the mycelial growth of *E. sacchari* EH036. Eleven of these showed significant inhibitory effects on mycelial growth, but the extent of inhibition varied among different agents. Difenoconazole showed the highest inhibition rate, with an EC_50_ = 0.00767 mg/L. It was followed by Propiconazole, Chlorobromoisocy-anuric acid, Trifloxystrobin·tebuconazole, Tebuconazole, and Myclobutanil, with EC_50_ less than 0.05 mg/L. In contrast, Zhongshengmycin, Pyrimethanil, Chlorothalonil, and Mancozeb demonstrated much weaker inhibitory activity against mycelial growth (Table 2).

**Table 2:** Inhibition of mycelial growth of *Elsinoë sacchari* by fungicides.

Fungicides	Toxicity equation	R^2^	EC_50_ (mg/L)	95% confidence intervals (mg/L)
Difenoconazole	y = 9.04 + 1.91x	0.988	0.00767	0.00656 ~ 0.00904
Propiconazole	y = 9.80 + 2.50x	0.963	0.012	0.011 ~ 0.014
Chlorobromoisocyanuric acid	y = 7.37 + 1.54x	0.986	0.0289	0.022 ~ 0.033
Trifloxystrobin·tebuconazole	y = 7.87 + 2.00x	0.995	0.0367	0.032 ~ 0.043
Tebuconazole	y = 6.91 + 1.37x	0.967	0.0403	0.032 ~ 0.049
Myclobutanil	y = 6.63 + 1.22x	0.991	0.0461	0.036 ~ 0.057
Pyraclostrobin·boscalid	y = 6.77 + 1.41x	0.990	0.0555	0.044 ~ 0.072
Pyraclostrobin	y = 6.55 + 1.28x	0.988	0.0615	0.048 ~ 0.077
Carbendazim	y = 10.81 + 5.71x	0.968	0.096	0.086 ~ 0.110
Thiophanate-methyl	y = 6.42 + 2.97x	0.954	0.333	0.261 ~ 0.531
Azoxystrobin	y = 5.28 + 0.69x	0.972	0.393	0.249 ~ 0.575
Zhongshengmycin	y = 3.88 + 0.42x	0.965	464.159	227.552 ~ 944.545
Pyrimethanil	y = 3.36 + 0.57x	0.828	753.690	354.308 ~ 1146.175
Chlorothalonil	y = 2.71 + 0.56x	0.843	12282.470	7474.278 ~ 23076.687
Mancozeb	y = 3.55 + 0.32x	0.888	33982.083	14295.609 ~ 95332.691

R^2^ = correlation coefficient; EC_50_ = median effect concentration.

Thirteen fungicides had a reasonable inhibitory effect on spore germination of EH036, with Pyraclostrobin·boscalid being the most potent with an EC_50_ = 0.000347 mg/L. Next were Trifloxystrobin·tebuconazole, Pyraclostrobin·boscalid, Mancozeb, Chlorothalonil, and Tebuconazole, with EC_50_ less than 0.1 mg/L. In contrast, Chlorobromoisocyanuric acid and Thiophanate-methyl were much less effective in the inhibition of spore germination (Table 3). When both mycelial growth and spore germination were considered, Trifloxystrobin·tebuconazole and Pyraclostrobin·boscalid emerged as the most balanced fungicides for inhibiting *E. sacchari*.

**Table 3:** Inhibition of spore germination of *Elsinoë sacchari* by fungicides.

Fungicides	Toxicity equation	R^2^	EC_50_ (mg/L)	95% confidence intervals (mg/L)
Pyraclostrobin·boscalid	y = 10.57 + 1.61x	0.998	0.000347	0.000288 ~ 0.000413
Trifloxystrobin·tebuconazole	y = 9.87 + 1.89x	0.996	0.00265	0.00228 ~ 0.00312
Pyraclostrobin	y = 5.97 + 0.81x	0.989	0.0635	0.0371 ~ 0.0837
Mancozeb	y = 6.75 + 1.52x	0.994	0.0706	0.0577 ~ 0.0811
Chlorothalonil	y = 6.55 + 1.41x	0.992	0.0796	0.0674 ~ 0.105
Tebuconazole	y = 6.92 + 1.82x	0.990	0.0881	0.0717 ~ 0.100
Difenoconazole	y = 5.26 + 0.83x	0.975	0.486	0.353 ~ 0.725
Zhongshengmycin	y = 5.11 + 1.01x	0.968	0.778	0.396 ~ 1.793
Propiconazole	y = 5.06 + 0.78x	0.996	0.838	0.566 ~ 1.393
Pyrimethanil	y = 4.81 + 1.78x	0.978	1.279	0.756 ~ 2.061
Carbendazim	y = 4.90 + 0.84x	0.968	1.315	0.962 ~ 1.865
Myclobutanil	y = 4.89 + 0.75x	0.981	1.402	0.946 ~ 2.212
Azoxystrobin	y = 4.85 + 0.97x	0.985	1.428	1.041 ~ 2.223
Chlorobromoisocyanuric acid	y = 3.83 + 0.49x	0.903	244.205	124.682 ~ 442.855
Thiophanate-methyl	y = 3.60 + 0.46x	0.961	1105.295	543.166 ~ 2124.703

R^2^ = correlation coefficient; EC_50_ = median effect concentration.

To further validate the inhibitory effect of the fungicides on *E. sacchari*, four randomly selected isolates (EH007, EH013, EH017, and EH032) were tested for sensitivity to Trifloxystrobin·tebuconazole, Myclobutanil, Pyraclostrobin·boscalid, and Thiophanate-methyl. Pyraclostrobin·boscalid showed the highest inhibition rate on the mycelial growth and spore germination of *E. sacchari*, followed by Trifloxystrobin·tebuconazole, Myclobutanil, Thiophanate-methyl (Supplementary Tables S2, S3). The inhibitory effects of each fungicide remained consistent across the different *E. sacchari* isolates, showing little inter-strain variation.

### 3.5 Field trials of fungicides for control of Sugarcane White Rash Disease

A field trial of SWRD with fungicide was conducted in Xichang Village, HePu County, Guangxi Province in 2023. Fungicides were selected based on their *in vitro* efficacy in inhibiting the pathogen’s mycelial growth and spore germination. Highly effective fungicides, Trifloxystrobin·tebuconazole and Pyraclostrobin·boscalid, as well as the moderately effective Myclobutanil and marginally effective Thiophanate-methyl, were included.

Initial SWRD symptoms appeared in the trial field in early May, and the disease index (DI) was approximately 15 by 6 June 2023, when the main sugarcane plants were at the 10-leaf stage, and the first spray was conducted. Two additional sprays were conducted consecutively with an interval of 7 days (the last spray on June 21 of 2023). The efficacy of the fungicides was evaluated 10 days after the last spray by comparing the DI, DIGR, and DRCE.

As shown in Table 4, the disease progressed rapidly in the control (CK), with DI increasing from 14.56 to 36.14 and a DIGR of 148.21%. In contrast, all fungicide treatments significantly reduced disease progression, with DIGRs ranging from −35.62% to 98.14%. The negative DIGRs were due to much fewer lesions on the newly born leaves after fungicide treatment. Among the treatments, Trifloxystrobin·tebuconazole at 0.250 g/L achieved the best control efficacy (DRCE = 74.07%), followed by Pyraclostrobin·boscalid at 0.190 g/L (DRCE = 68.96%). At the same time, Thiophanate methyl at 0.700 g/L recorded the poorest performance (DRCE = 19.87%). Thus, Pyraclostrobin·boscalid and Trifloxystrobin·tebuconazole achieved the best control results with DRCE from 64.38% to 74.07%, while Myclobutanil (DRCE = 37.90%–46.94%) and Thiophanate-methyl (DRCE = 19.87%–29.48%) were less desirable (Table 4).

**Table 4:** The control effect of fungicides on sugarcane white rash disease.

Fungicide	DI_before_	DI_after_#	DIGR (%)	DRCE^z^ (%)
Trifloxystrobin·tebuconazole (0.250 g/L)	15.72 ± 0.95a	10.12 ± 0.58e	−35.62	74.07 ± 0.59a
Trifloxystrobin·tebuconazole (0.188 g/L)	13.29 ± 1.32bc	9.19 ± 1.01e	−30.85	72.11 ± 2.05a
Pyraclostrobin·boscalid (0.190 g/L)	14.01 ± 1.51abc	10.75 ± 0.74e	−23.27	68.96 ± 2.74a
Pyraclostrobin·boscalid (0.253 g/L)	15.74 ± 0.80a	13.91 ± 0.81d	−11.63	64.38 ± 1.97a
Myclobutanil (0.0833 g/L)	14.98 ± 0.78ab	19.58 ± 3.75c	30.71	46.94 ± 12.90b
Myclobutanil (0.0625 g/L)	13.02 ± 0.82c	20.02 ± 2.39c	53.76	37.90 ± 8.69c
Thiophanate-methyl (0.933 g/L)	14.87 ± 0.13abc	26.05 ± 1.26b	75.18	29.48 ± 2.83d
Thiophanate-methyl (0.700 g/L)	14.49 ± 0.89abc	28.71 ± 1.36b	98.14	19.87 ± 8.56d
CK	14.56 ± 1.20abc	36.14 ± 1.09a	148.21	/

^#^10 days after the third spray of fungicide. ^z^Values are means and standard errors of multiple observations from 3 replicate plots. Columns with the same letters mean no significant difference according to Duncan’s test (*p* < 0.05). DI = disease index; DI_before_ = DI before application of the fungicide; DI_after_ = DI after application of the fungicide; DIGR = disease index growth rate; DIGR_control_ = DIGR in control area; DRCE = disease relative control efficacy.

The impact of SWRD on sugarcane agronomic traits was also investigated at plant maturity. Significant differences in plant height were observed among fungicide-treated plants. As shown in Table 5, plant height, stalk diameter, and cane yield were significantly increased after application of fungicides Trifloxystrobin·tebuconazole and Pyraclostrobin·boscalid. Impressively, an increase of 14.25% in cane yield was obtained in treatments with Trifloxystrobin·tebuconazole at 0.250 g/L or Pyraclostrobin·boscalid at 0.190 g/L. However, application of fungicides did not consistently improve the stalk diameter or sugar content (brix) (Table 5). These results suggest that SWRD may have a limited influence on sugar accumulation but can reduce yield by suppressing plant height.

**Table 5:** Impact of sugarcane white rash disease on agronomic traits of sugarcane*.

Fungicide	Plant height^z^ (cm)	Stalk diameter^z^ (mm)	Stalk weight^z^ (kg)	Brixz (%)	Yield (t·hm^−2^)	Yield increase (%)
Trifloxystrobin·tebuconazole (0.250 g/L)	303.88 ± 1.24ab	28.09 ± 0.16a	2.07 ± 0.045a	20.33 ± 0.18bc	124.30a	14.25
Pyraclostrobin·boscalid (0.190 g/L)	304.92 ± 1.64a	28.11 ± 0.06a	2.07 ± 0.068a	20.48 ± 0.21bc	124.30a	14.25
Trifloxystrobin·tebuconazole (0.188 g/L)	301.00 ± 3.16bc	28.11 ± 0.20a	2.03 ± 0.048ab	20.67 ± 0.12ab	121.65ab	11.81
Pyraclostrobin·boscalid (0.253 g/L)	302.75 ± 1.08ab	28.01 ± 0.07a	2.02 ± 0.072ab	20.19 ± 0.18c	121.00ab	11.21
Myclobutanil (0.0833 g/L)	298.57 ± 1.05 cd	28.06 ± 0.22a	1.93 ± 0.040bc	20.18 ± 0.10c	115.95bc	6.57
Thiophanate-methyl (0.933 g/L)	297.50 ± 2.13d	28.21 ± 0.02a	1.89 ± 0.073 cd	20.63 ± 0.15ab	113.10 cd	3.95
Myclobutanil (0.0625 g/L)	297.43 ± 2.63d	28.20 ± 0.10a	1.87 ± 0.021 cd	20.94 ± 0.30a	112.05 cd	2.99
Thiophanate-methyl (0.700 g/L)	296.02 ± 0.46d	28.10 ± 0.18a	1.85 ± 0.059 cd	20.07 ± 0.40c	111.00 cd	2.02
CK	297.07 ± 0.10d	28.25 ± 0.12a	1.81 ± 0.037d	20.42 ± 0.22bc	108.8d	/

^*^Traits were determined on 23 November 2023. ^z^Values are means and standard errors of multiple observations from three replicates. Columns with the same letters mean no significant difference according to Duncan’s test (*p* < 0.05).

## 4 Discussion

Endemic to SWRD occurs only in the coastal area of Guangxi province, China, although sporadic occurrences have been observed in inland areas. However, the specific meteorological parameters that favor disease development have yet to be identified to fully understand the disease dynamics. In this study, *E. sacchari* was identified as the pathogen responsible for SWRD in Guangxi, China, based on evidence from pathogenicity tests, symptoms on sugarcane, and morphological characteristics. Since no molecular sequences of *E. sacchari* were previously available in public databases, phylogenetic analysis revealed that this species is most closely related to *E. arachidis* (Fan et al., 2017; Jayawardena et al., 2015). This study presents the first deposition of molecular sequences (ITS, LSU, RPB2, and TEF1-α) of *E. sacchari* to the GenBank database. The fungus grew much better at 20–30 °C *in vitro*. This coincides with the endemic season of June to August in the coastal region. Similarly, other diseases caused by *Elsinoë* species, such as citrus scab and grape black pox, are also known to be more severe in hot and humid regions (Agostini et al., 2003; Carisse et al., 2024).

Production of conidia has long been a challenge for *Elsinoë* species (Li et al., 2018; Masheti Y. O. et al., 2024). Current protocols of producing conidia for the citrus scab pathogen *Elsinoë* spp. are all based on rainwater induction of *Elsinoë* mycelium (Hyun et al., 2015; Whiteside, 1975). However, *E. sacchari* failed to produce conidia using these methods. In this study, however, *E. sacchari* was found to produce hyaline conidia on OA plate, suggesting a varied mechanism of sporulation regulation. The efficient conditioning method here will facilitate both basic research and applied study of *E. sacchari* in the future.

Elsinochrome (ESC) is a perylenequinone-type mycotoxin produced by many *Elsinoë* species, and acts as a key virulence factor in the pathogenicity of this genus (Gonsalves et al., 2026; Hao et al., 2026). This toxin is red or orange and can inhibit the photosensitivity of the host and non-host plant by generating reactive oxygen species under light (Jiao et al., 2019, 2021). It was reported that the synthesis of ESC in *E. arachidis* was light-dependent (Jiao et al., 2019; Liu et al., 2024). Interestingly, the red pigment produced by *E. sacchari*, presumably the ESC, appeared to be light insensitive, implying that varied ESC regulation mechanisms exist between *E. arachidis* and *E. sacchari*. Since the primary objective of this study was fungicide screening and field control trials, ESC was not investigated further.

*Elsinoë* fungi infect the leaves and fruits of various crops, leading to significant losses (Henriques et al., 2026; Paudyal et al., 2017). Currently, control of diseases caused by *Elsinoë* fungi primarily relies on chemical agents (Pedrotti et al., 2022). For instance, Chlorothalonil had been used as a preventive agent against grape black pox (Magarey et al., 1993) and Carbendazim for bean scab (Masheti Y. et al., 2024). In our *in vitro* assays, however, these two fungicides showed limited activity against mycelium growth or conidial germination of *E. sacchari*. Among the 15 fungicides tested, triazole fungicides showed the best effects in inhibiting mycelial growth and spore germination. Triazole fungicides primarily disrupt the synthesis of ergosterol, which influences fungal cell wall formation (Khalil et al., 1990; Lupetti et al., 2002). It was also observed that conidia were more sensitive than hyphae to the same potent fungicides in *E. sacchari*. Therefore, an ideal fungicide for SWRD control should inhibit both spore germination and hyphal growth with a low EC_50_.

The EC₅₀ values of Trifloxystrobin·tebuconazole, Myclobutanil, Pyraclostrobin·boscalid, and Thiophanate-methyl showed minimal variation across all tested isolates of *E. sacchari*. This consistent sensitivity suggests an absence of pre-existing resistance in the population and provides a strong rationale for evaluating these fungicides in field trials (Song et al., 2016). Accordingly, Trifloxystrobin·tebuconazole and Pyraclostrobin·boscalid demonstrated satisfactory efficacy in the field trials for SWRD control.

## 5 Conclusion

*E. sacchari* was identified as the pathogen of SWRD in Guangxi, China, based on host range, symptoms, and morphological characteristics. Key biological traits of the fungus were characterized. Several fungicides that showed high efficacy against *E. sacchari* were screened in vitro and confirmed by field trials. In particular, Trifloxystrobin·tebuconazole and Pyraclostrobin·boscalid are recommended for the control of SWRD in sugarcane.

## Data Availability

The datasets presented in this study can be found in online repositories. The names of the repository/repositories and accession number(s) can be found in the article/Supplementary material.
